# Seasonal and Ontogenetic Changes in Movement Patterns of Sixgill Sharks

**DOI:** 10.1371/journal.pone.0012549

**Published:** 2010-09-08

**Authors:** Kelly S. Andrews, Greg D. Williams, Phillip S. Levin

**Affiliations:** Northwest Fisheries Science Center, National Oceanic and Atmospheric Administration, Seattle, Washington, United States of America; Institut Pluridisciplinaire Hubert Curien, France

## Abstract

**Background:**

Understanding movement patterns is fundamental to population and conservation biology. The way an animal moves through its environment influences the dynamics of local populations and will determine how susceptible it is to natural or anthropogenic perturbations. It is of particular interest to understand the patterns of movement for species which are susceptible to human activities (e.g. fishing), or that exert a large influence on community structure, such as sharks.

**Methodology/Principal Findings:**

We monitored the patterns of movement of 34 sixgill sharks *Hexanchus griseus* using two large-scale acoustic arrays inside and outside Puget Sound, Washington, USA. Sixgill sharks were residents in Puget Sound for up to at least four years before making large movements out of the estuary. Within Puget Sound, sixgills inhabited sites for several weeks at a time and returned to the same sites annually. Across four years, sixgills had consistent seasonal movements in which they moved to the north from winter to spring and moved to the south from summer to fall. Just prior to leaving Puget Sound, sixgills altered their behavior and moved twice as fast among sites. Nineteen of the thirty-four sixgills were detected leaving Puget Sound for the outer coast. Three of these sharks returned to Puget Sound.

**Conclusions/Significance:**

For most large marine predators, we have a limited understanding of how they move through their environment, and this clouds our ability to successfully manage their populations and their communities. With detailed movement information, such as that being uncovered with acoustic monitoring, we can begin to quantify the spatial and temporal impacts of large predators within the framework of their ecosystems.

## Introduction

How animals move through their environment influences the dynamics of local populations, genetic variability, and the re-colonization of depauperated habitats. Patterns of movement are thus fundamental to population dynamics and conservation biology [1]. Patterns of movement can also affect how susceptible local populations may be to perturbations such as climate change, disease, exploitation, pollution, or habitat loss [2]–[4]. The magnitude of impact of a predator on community structure is also clearly influenced by how predators move across space [5]. Indeed, in order to manage populations and the communities within which they reside, we need to have some understanding of patterns of movement [6]–[8].

Movement is generally studied at three different temporal scales: daily, seasonally, and ontogenetically. Most species have consistent diel activity patterns, with some species more mobile during the day (e.g. grizzly bears [9], butterflies [10], and parrotfish [11]), while others are more mobile during the night (e.g. fruit bats [12], mountain lions [13], striped skunks [14]). Patterns of movement also vary seasonally, but the mechanisms underlying seasonal movements vary among taxa. For example, populations of wildebeest *Connochaetes spp*, zebra *Equus zebra*, and Thompson's gazelle *Eudorcas thomsoni* undergo epic migrations across the African savanna in search of suitable grazing lands [15]. Similarly, patterns of carnivore movement, such as the wolf *Canis lupus*, are influenced by their seasonally migrating prey (e.g. caribou *Rangifer tarandus*) [16]. Although the mechanisms are unknown, white sharks *Carcharodon carcharias* show seasonal patterns of movement along the coast of Australia [17], South Africa [18], and seasonal inshore/offshore movements in California, USA [19]. Some species also display seasonal patterns of movement when they are exposed to thermal stress; for instance, the West Indian manatee *Trichecus manatus* moves north along the eastern coastline of North America in the summer as temperatures warm and then return to southern waters in the winter when temperatures begin to decline [20].

Patterns of movement can also vary with ontogeny. In marine environments, it is common for adult invertebrates and fish to leave relatively narrow home ranges for distant spawning grounds [21]. For example, mature Nassau grouper *Epinephelus striatus* can migrate >200 km to spawning grounds each winter [22], and mature female blue crabs *Callinectes sapidus* migrate from low salinity inlets of Chesapeake Bay to the mouth of the estuary each fall [23], [24]. Many marine species are also characterized by life histories in which young-of-year and juveniles live in habitats that are relatively protected from predators. For example, young lemon sharks *Negaprion brevirostris* in the Bahamas and Florida, USA restrict their movements to shallow seagrass flats and shoreline mangroves within the lagoons, effectively reducing their risk to potential predators [25], [26]. In addition, older life-history stages of many shark species occupy different habitats than juveniles [27], [28]

Quantifying movement of marine species, especially those that are highly mobile or deep dwelling has historically been extremely difficult; however, technological improvements in satellite and acoustic telemetry have made it possible for researchers to quantify the spatial and temporal patterns of movement of such species. Of particular interest are those species that are vulnerable to human activities (e.g. fishing) or that exert a large influence on community structure, such as sharks. Studies investigating diel patterns show movement patterns that vary by species: many species occupy deeper waters during the day and move closer to shore or to the surface at night [29]–[34], while others do not display this behavior [35]–[38]. Seasonal movements by pelagic shark species appear common [39]–[41]. For example, salmon sharks *Lamna ditropis* display seasonal movements between subarctic foraging grounds in the summer to subtropical destinations in the spring [42], [43]. However, there have been relatively few studies investigating the patterns of movement of benthic sharks [33], [37], [44]–[47] and none of these were performed long enough to capture seasonal patterns of movement for individuals. Studies using mark-recapture methods [48] and acoustic tracking [27] have shown sharks of different life-history stages use different habitats, but we are unaware of studies that have monitored the same individual over time and captured ontogenetic movements from juvenile “nursery” habitat to adult habitat or potential mating grounds.

In Puget Sound, (Washington state, USA) sixgill sharks *Hexanchus griseus* display diel patterns of movement in which they inhabit deeper waters during the day and shallower waters during the night [34]. Pilot studies suggest sixgills occupy localized areas of Puget Sound during the fall with average daily movement between 0.2 to 3.1 km and maximum displacement from tagging sites between 8.4 to 29.2 km [47]. In addition, these metrics were negatively related to size, such that larger sixgills appear to move less than smaller sixgills. Smaller sixgills, however, were detected nearly eight times as often as larger sixgills [47]. This potential discordance between movement and detectability could be due to larger sharks moving out of the range of the acoustic array used in previous studies. Using a larger scale array over a longer period of time could test whether this pattern is real or whether there are seasonal patterns that obscure this relationship.

In this study, we use two large-scale integrated arrays of passive acoustic receivers to monitor the broad-scale spatial patterns of movement of sixgill sharks inside and outside Puget Sound from 2006–2009. Specifically, we address three main questions in this paper. First, we ask how sedentary sixgill sharks are and if variability in the degree of sedentariness can be explained by traits of individual sharks or the time scale over which this question is asked? Secondly, we ask if sixgill sharks show any seasonal patterns in either location or rate of movement within Puget Sound? Third, we ask whether sixgill sharks leave Puget Sound, and if so, is this related to their size or gender?

## Methods

### Study location

Puget Sound is a highly urbanized inland estuary of the eastern North Pacific Ocean in Washington, USA. Relatively shallow sills isolate the main basin from the other sub-basins within Puget Sound, which can potentially restrict ocean circulation and the movement of organisms, sediments and contaminants [49]. Tides, gravitational forces and seasonal freshwater input drive estuarine circulation in Puget Sound. Within the main basin, Elliott, Commencement, and Port Gardner Bays, associated with Seattle, Tacoma, and Everett, respectively, have depths in excess of 100 m. The average depth of greater Puget Sound is 62.5 m at mean low tide, while the main channel is ∼250 m at its greatest depth. The main basin is generally stratified in the summer, because of river discharge and solar heating, and is often well mixed in the winter [49]. This seasonal pattern is responsible for a peak in production of phytoplankton and macroalgae during the summer in Puget Sound [50], which influences the abundance of consumers and predators in the pelagic and benthic communities [51].

### Study species

The sixgill shark is one of the largest predatory sharks in the world with total lengths up to 485 cm and is the largest resident fish in Puget Sound. Sixgill sharks are typically demersal and found in deep water along the continental shelf and upper slope; however, they may also move into shallow waters, and juveniles frequent nearshore waters [34], [47], [52], [53]. In British Columbia, Canada, the abundance of immature sixgill sharks is greater during the day in summer months relative to other months of the year [54].

Sixgill sharks are ovoviparous with litters ranging between 22 and 108 pups [52]. Males appear to mature at approximately 3.1 m total length, while females mature at nearly 4.2 m [53], [55]. Little is known, however, about age at maturity or size at age of individuals. Growth rates of sixgill sharks are relatively unknown, although captive young-of-year nearly doubled in size during their first year [53] and one recaptured sub-adult in Puget Sound grew at a rate of 12 cm/yr [34]. Sixgill sharks feed on a wide variety of prey including other sharks, rays, pelagic and demersal teleosts, marine mammals and whale carrion [53], [56].

### Collecting and tagging sharks

**Figure 1:**
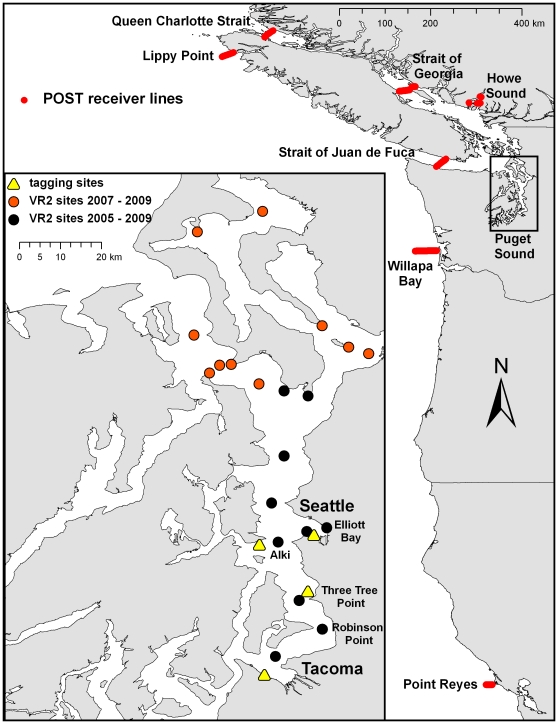
Locations of passive acoustic receivers from two large-scale integrated arrays. The Pacific Ocean Shelf Tracking array occurs as lines of receivers along the west coast of North America, while the inset shows the location of the primary acoustic receivers used in this study within Puget Sound, WA.

Collection and tagging of sharks during this study was approved and performed according to regulations provided by the Washington State Department of Fish & Wildlife Scientific Collection Permits #07-349, #06-397, and #05-330. We collected a total of 70 sixgill sharks in Puget Sound between November 2005 and April 2008 using standard baited longline methods [34], [57]. Of those 70, we tagged 39 with pressure sensor acoustic transmitters. Twenty-two sharks were collected in Elliott Bay, ten near Three Tree Point, five at the south end of Bainbridge Island, and two near Commencement Bay (Fig. 1). Basic biological information for each shark is described in Table 1. Upon capture, sharks were brought on board and their gills irrigated with seawater. We measured, weighed and sexed each shark, and placed an external Floy© tag through the dorsal fin. We implanted one Vemco V16P coded acoustic transmitter with pressure sensor into the midline of the peritoneal cavity via a 3-cm incision at the anterior end of the pelvic fins. After the incisions were sutured, sharks were returned to the water. All sharks were handled the same and time out-of-water was similar for all individuals (range: 5–10 minutes).

**Table 1:** Biological data of 39 sixgill sharks tagged with acoustic transmitters from 2005–2008 in Puget Sound, WA, USA.

Shark ID	Tagging Date	Tagging Location	Total length (cm)	Weight (kg)	Gender
4083	23 Jun 2005	Tacoma	238	.	M
4084	23 Jun 2005	Tacoma	200	.	F
25	16 Nov 2005	Three Tree Point	182	38	M
26	16 Nov 2005	Three Tree Point	204	60	F
27	16 Nov 2005	Three Tree Point	181	35	M
28	16 Nov 2005	Three Tree Point	176	33	M
29	16 Nov 2005	Three Tree Point	205	39	M
30	16 Nov 2005	Three Tree Point	140	13	F
31	16 Nov 2005	Three Tree Point	109	6	F
32	16 Nov 2005	Three Tree Point	225	72	F
33	16 Nov 2005	Three Tree Point	193	21	F
199	16 Nov 2005	Three Tree Point	168	23	M
190	4 May 2006	Elliott Bay	240	105	F
198	4 May 2006	Elliott Bay	203	53	F
200	4 May 2006	Elliott Bay	237	92	M
201	4 May 2006	Elliott Bay	285	173	M
202	4 May 2006	Elliott Bay	269	144	M
203	4 May 2006	Elliott Bay	203	50	F
204	4 May 2006	Elliott Bay	270	137	M
207	4 May 2006	Elliott Bay	293	115	F
78	21 Jan 2007	Elliott Bay	220	75	F
79	20 Mar 2007	Elliott Bay	276	126	F
81	20 Mar 2007	Elliott Bay	248	90	M
82	20 Mar 2007	Elliott Bay	245	94	M
83	20 Mar 2007	Elliott Bay	233	101	F
80	16 Apr 2007	Elliott Bay	183	33	M
84	16 Apr 2007	Elliott Bay	150	20	M
85	16 Apr 2007	Elliott Bay	202	66	M
86	16 Apr 2007	Elliott Bay	218	65	F
87	14 May 2007	Bainbridge	175	25	F
88	16 May 2007	Elliott Bay	154	17	F
89	12 Jun 2007	Bainbridge	248	83	F
90	28 Aug 2007	Elliott Bay	280	151	F
195	28 Aug 2007	Elliott Bay	250	91	F
1031	10 Oct 2007	Bainbridge	237	68	M
1034	11 Oct 2007	Bainbridge	230	65	M
1035	12 Oct 2007	Bainbridge	261	99	F
148	9 Apr 2008	Elliott Bay	247	91	M
149	9 Apr 2008	Elliott Bay	241	114	F

The coded transmitters emit a train of ‘pings’ at 69 kHz randomly every 40–114 seconds that contains a specific ID code and a value of the sensor's depth in the water which allows users to identify individuals and collect three-dimensional data. The life span of the transmitters was 1429 days for ID codes 25–33, 78–90, and 148–149, 894 days for ID code 190, 561 days for ID code 195, 1039 days for ID codes 198–204, and 207, 952 days for ID codes 1031, 1034, and 1035, and 1904 days for ID codes 4083 and 4084. The coded transmitters were detected by passive acoustic receivers deployed in large-scale arrays throughout Puget Sound and the Georgia Basin.

### Integrated acoustic receiver arrays

In this study, we took advantage of two large-scale passive acoustic monitoring arrays that use Vemco© acoustic receivers: VR2s, VR2Ws or VR3s. In Puget Sound, a consortium of city, state, federal, and native tribal agencies has developed an acoustic array of single receivers located throughout Puget Sound. The data collected by this consortium of researchers are managed by an online database known as HYDRA – HYdrophone Data Repository [http://hydra.sounddatamanagement.com/]. Users upload acoustic receiver data to HYDRA for query by anyone in the consortium. At present, this database contains more than 12 million detections of known and unknown acoustic transmitters from a total of 693 receiver deployments. The timing and duration of receiver deployments varies and is dependent on the goals of individual researchers. Our research group, however, has maintained a backbone of 10 receivers that have been deployed throughout central Puget Sound from December 2005–October 2009 and a second core group of 10 receivers deployed in Admiralty Inlet and Whidbey Basin from April 2007–May 2009 (Fig. 1). All receivers continuously ‘listened’ for Vemco© acoustic transmitters throughout the duration of their deployment. The V16P transmitters used in this study have a power output of 160 dB and an estimated radius of detection with Vemco VR2 receivers of ∼800 m [58]. When transmitters are detected, a date/time stamp and depth-of-transmitter value is stored in memory and then retrieved upon download of the receiver.

In addition to the array in Puget Sound, the Pacific Ocean Shelf Tracking (POST) Project maintained several “lines” of acoustic receivers in the Georgia Basin, Puget Sound, and off the coast of North America from Alaska to California (Fig. 1). Within a line, receivers are spaced approximately 800 m apart. This spacing forms a gate across some channels of water (such as the Strait of Juan de Fuca) or extends from shore towards the continental shelf. The V16P transmitters used in this study were detected simultaneously at multiple receivers on these lines, which supports the likelihood these lines provided nearly 100% detection of our sharks through these bodies of water [59]. Four of these lines were deployed for nearly the entire duration of our study (Strait of Juan de Fuca: April 2006 – end of study; the northern Strait of Georgia, BC: April 2005 – end of study; Queen Charlotte Strait, BC: April 2006 – end of study; and Lippy Point, BC: June 2006 – end of study). There were also gates across Howe Sound, BC from April–July in 2006, 2007 and 2008. Detections of sixgill sharks also occurred at lines at Point Reyes, CA, which extended 12 km from the shore and was deployed from July 2008 through the end of our study, and at Willapa Bay, WA, which extended 35 km from the shore to the edge of the continental shelf. POST also manages a database which can be queried by individual researchers for detections of their transmitters.

For data analysis purposes, we classify the location of receivers in Puget Sound to a site and a region, while we classify receivers in the POST array to a single line name. A “site” includes detections for one receiver, whereas a “region” consists of all receivers within a 7-km diameter circle, generally located around one of our “backbone” receivers (Fig. 1). We chose 7 km as the diameter of a region based on the average mean displacement found in sixgill sharks in Puget Sound during previous work [47].

### Data analysis

Prior to analyses, we reduced our dataset in two ways. First, we used detections from HYDRA only when there were at least three detections of the transmitter at the same site within a ten-minute period to eliminate potential false detections. Second, we reduced our data to unique date-site detections of sharks, such that a shark is either present or absent at a site on each date. This procedure standardized the frequency of sampling and helped remove serial autocorrelation from the dataset [1].

In order to test whether sharks were detected more frequently based on size, gender or tagging location, we calculated the “detectability” of each shark by dividing the number of days a shark was detected by the number of days the shark was at liberty in Puget Sound. We used detectability as the dependent variable in a linear mixed model (PROC MIXED, [60]) with total length at capture of shark (TL; cm), gender and the interaction between TL and gender as fixed effects with tagging location as a random effect. Interaction terms for all analyses were iteratively removed from the model if corresponding p-values were >0.25 [61]. We also performed cluster analysis [62] using detectability to assess whether patterns exist in the temporal interval of detection. In other words, we wished to determine if some groups of sharks were detected on a frequent basis, while other groups of sharks were detected less frequently. We used Euclidean distance to determine clusters.

**Sedentariness of sharks:** We define sedentariness as the proportion of time that a shark was detected in the same location as its previous detection. We calculated this metric as follows:
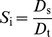
where S_i_ is sedentariness for each shark, D_s_ is the number of days each shark was detected in the same site (or region) as its previous detection, and D_t_ is the total number of detection days for each shark (e.g. How often is a shark in the same location?). We then used this metric as the dependent variable in a linear mixed model to test whether sedentariness was related to gender, TL at capture or tagging location. We then expanded this metric to calculate the proportion of time a shark was detected at the same site at some lag time in the future (e.g. How often will a shark be in the same location in 30 days?). We calculated this metric by dividing the total number of detection days for each shark (D_t_) by the number of days it was detected in the same location at the given lag period. We calculated these values for both site and region over a range of lag times between 1 day and 365 days.

**Seasonal patterns of movement in Puget Sound:** To investigate seasonal patterns of movement within Puget Sound, we excluded detections from the POST array for the following analyses. We calculated the location of each shark detection as the distance (km) between a shark's tagging site and the receiver where the shark was detected on a north/south scale (i.e. negative values meant movement to the south of tagging site). This allowed us to standardize movements of sharks independent of their tagging location. We calculated the mean (± SE) distance from tagging site for each shark for each month. Monthly means were used as the dependent variable in a linear mixed model with month, gender, TL at capture, and each of the two-way interactions as fixed effects and shark as a random effect.

In order to test whether the rate at which sharks move throughout Puget Sound varied over the year, we calculated the mean (± SE) rates of movement between detections for each month during the study period. Monthly means were calculated by summing the cumulative distance moved each month and dividing by the time between first and last detection of each month. Monthly rate of movement for each shark was used as the dependent variable in a linear mixed model with month, gender, TL at capture, and each of the two-way interactions as fixed effects and shark as a random effect.

**Ontogenetic shift out of Puget Sound:** We queried the POST database for detections of tagged sixgill sharks. We calculated distances traveled and rates of movement from Puget Sound to these lines of receivers. It is important to note that these “gates” across the Straits of Juan de Fuca and Georgia were deployed for nearly the entire time our sharks were at liberty. The one break in coverage at Strait of Juan de Fuca (October 2005 to April 2006) occurred when all sharks tagged at the time were accounted for inside Puget Sound. There were instances of faulty receivers that may have created large enough gaps in the lines to allow a shark to move through undetected, but these were very infrequent events. Thus, if one of our sixgill sharks migrated through the Straits of Juan de Fuca or Georgia, there were very limited periods of time and space it would have escaped detection.

**Figure 2:**
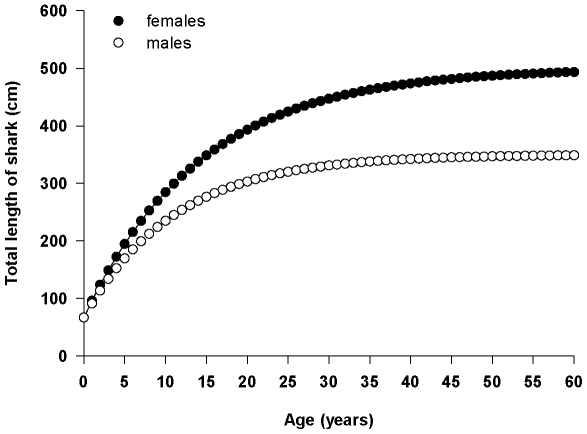
Growth curves of male and female sixgill sharks.

In order to determine if there were ontogenetic shifts in movement, we needed an estimate of shark size when they left the Puget Sound region. We thus estimated growth rates predicted from the growth curves (see below; Fig. 2) to calculate the total length of each shark when it was last detected in Puget Sound or when it was first detected outside Puget Sound. Sharks were then classified by whether they left Puget Sound or stayed in Puget Sound. This “status” of shark was used as the dependent variable in a logistic regression (Systat 11.0) with calculated TL of shark as the predictor variable. We ran this model for each gender separately. If TL was a significant predictor variable, we calculated the probability of a shark leaving Puget Sound using the logistic regression function:
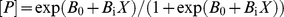
where [*P*] is the probability of a shark leaving Puget Sound, *B*_0_ is the constant coefficient, *B*_1_ is the variable's coefficient, and *X* is a range of values of TL [63].

Growth curves were estimated for male and female sixgill sharks following the standard von Bertalanffy growth model
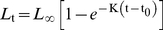
where *L*_t_ is the predicted length (cm) at age t; *L*_∞_ is equal to the mean theoretical maximum total length; *K* is a growth rate parameter (per yr); and t_0_ is the theoretical age (yr) at zero length. We estimated *L_∞_* for males (350 cm) and females (500 cm) based on their maximum reported total lengths (350 cm and 485 cm, respectively; [53]). To solve *K*, we relied upon the theory that “there is an intrinsic inverse relationship between L_∞_ and *K*…” [64]. We used the growth parameter table in FISHBASE [www.fishbase.org] to collect estimates of L_∞_ and *K* for all studies which reported *L*_∞_, *K*, and *t*_0_ for non-captive shark species. This resulted in 130 studies from 40 different species, across 9 different families of sharks. We then regressed L_∞_ and *K* (r^2^ = 0.265, t = −6.786, p<0.001), and used parameters from this regression and our estimates of L_∞_ for males and females to solve for *K* (males: 0.09; females: 0.07). Finally, we solved for *t*_0_ using
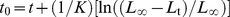
where *L*_t_ is a known length at age (size at birth) [65]. The *t*_0_ values were calculated from an average size at birth of 68 cm TL for both genders [53]. The calculated growth curves (Fig. 2) are theoretical and coarse, but they produce values which correspond closely with the one available growth rate (∼12 cm/year) from a recaptured 248-cm female sixgill shark [34], and allow us to roughly estimate the size of fish leaving the Sound.

In order to test whether sixgills changed their behavior prior to leaving Puget Sound, we compared the rates of movement of each shark during the three-month period before they left Puget Sound with the same three-month period during previous years. We used a three-month period because it roughly corresponds to the length of each season. Thus, we compared movements of sharks during the “season” in which it left Puget Sound to the same “season” as last year. We calculated each shark's rate of movement across these three-month periods and used these values in a paired t-test. We had enough data on seven sharks to perform this analysis.

## Results

**Figure 3:**
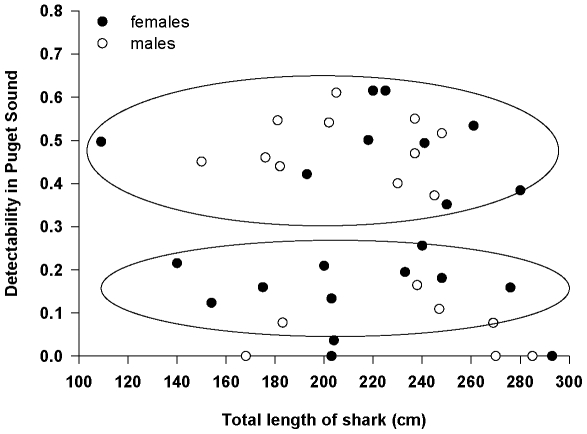
Frequency of detection of sixgill sharks in Puget Sound, WA, USA. Clusters show sharks detected at three different scales. The third cluster of six sharks is not circled because it interferes with the legibility of the x-axis.

There were 625,224 detections of the 39 tagged sharks during the four years of our study. We reduced the data set to 12,349 unique shark-date-site detections. Three sharks were detected for 3+ years, ten sharks for 2+ years, six for 1+ years, fifteen sharks for less than one full year, and five sharks were never detected. Cluster analysis revealed three groups based on their detectability (Fig. 3). Sharks in the largest group were detected on half of their days at liberty (n = 20; mean: 0.49; range: 0.35–0.62), the second group of sharks was detected only 16% of their days at liberty (n = 13; mean: 0.16; range: 0.08–0.26), while the third group was basically never detected (n = 6; mean: 0.01; range: 0.0–0.04). Linear mixed model results with and without this grouping factor did not reveal any significant differences in the detectability of sharks based on total length (w/group: F_1, 30_ = 0.26, p = 0.612; w/o group: F_1, 32_ = 1.65, p = 0.208), gender (w/group: F_1, 30_ = 0.05, p = 0.824; w/o group: F_1, 32_ = 1.70, p = 0.202), or the interaction between total length and gender (w/group: F_1, 30_ = 0.00, p = 0.947; w/o group: F_1, 32_ = 1.52, p = 0.227), nor was tagging location significant as a random effect (estimates were 0 in all runs of the model).

### Sedentariness of sharks

**Figure 4:**
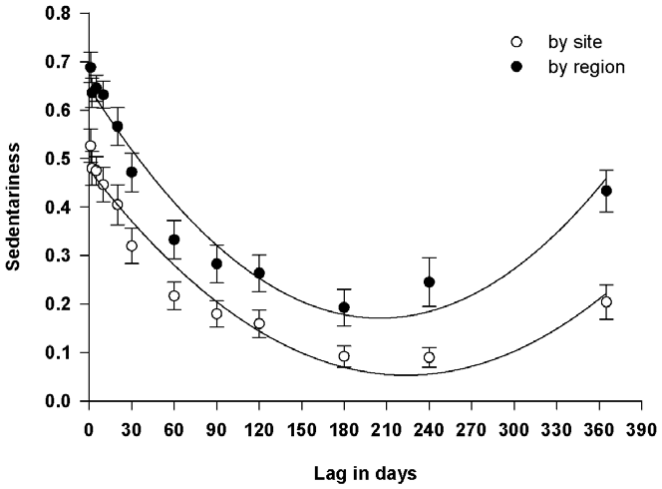
Sedentariness of sixgill sharks within Puget Sound, WA, USA. Sedentariness is the proportion of days a shark is detected at the same site (or region) at various lags of time from any point in the monitoring data. The parabolic relationship shows an annual pattern in which sixgills are sedentary within a site (or region) for weeks at a time, move slowly away to a second core area after six months, and then return to the original core area at the same time each year.

Of the 34 sharks detected, sharks were at the same location as the previous date of detection (sedentariness) 62%±3% SE of the time for site and 71%±3% SE of the time for region. There was no significant differences in sedentariness for TL (site: F_1, 27_ = 0.18, p = 0.677; region: F_1, 27_ = 0.19, p = 0.663), gender (site: F_1, 27_ = 1.38, p = 0.250; region: F_1, 27_ = 1.51, p = 0.230), or the interaction term (site: F_1, 27_ = 1.55, p = 0.224; region: F_1, 27_ = 1.72, p = 0.200), nor was tagging location significant as a random effect (estimates were 0 for site and region ). Next, we asked how often will a shark be in the same location in 1, 10, 30, 60…365 days. We detected a distinct parabolic relationship between sedentariness and lag period at both spatial scales (Fig. 4; site: r^2^ = 0.955; region r^2^ = 0.951). Sharks inhabit the same region for nearly 30 days before sedentariness drops below 50%. Sedentariness decreases until ∼180 days, and then increases to 43% by 365 days. Thus, sharks were more likely to be detected at the same site (or region) after one full year than they would be after 6 months. We also grouped sharks by their frequency of detection (50% and 16%), and saw the same parabolic relationship even for sharks detected at a much lower frequency (Sharks detected 50% of days at liberty: site: r^2^ = 0.927, region r^2^ = 0.923; Sharks detected 16% of days at liberty: site: r^2^ = 0.885; region r^2^ = 0.848).

### Seasonal patterns of movement in Puget Sound

**Figure 5:**
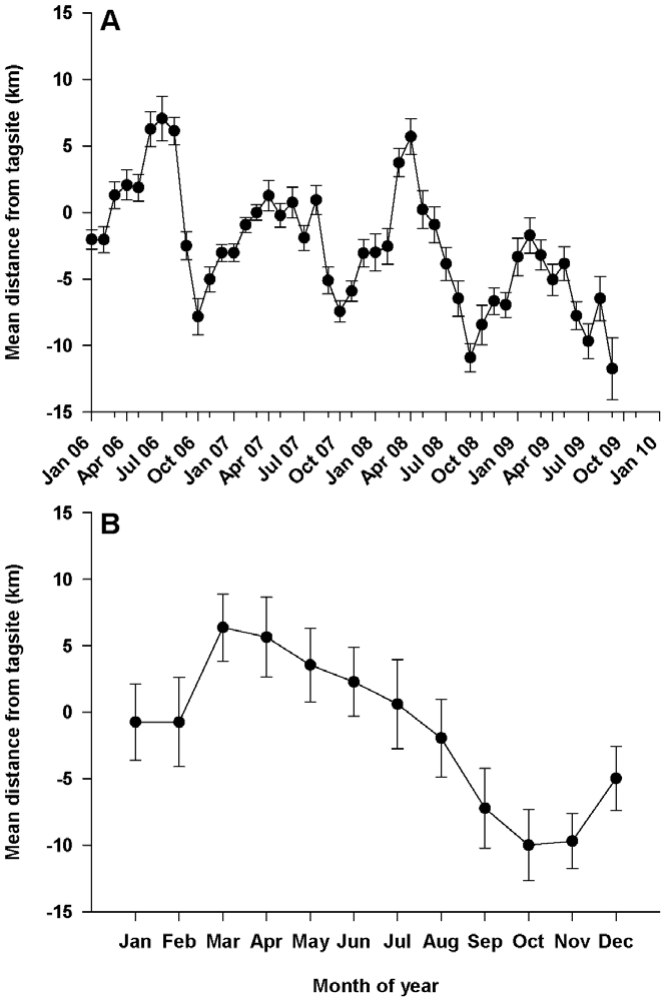
Mean distance from tagging location (km) of sixgill sharks in Puget Sound, WA, USA. Values were calculated across all sharks for A) each month from 2006 to 2009, and for B) each month across all years.

Sixgill sharks showed relatively consistent patterns across all four years in which they moved northward (increasing values) and were at their most northern locations in early spring or summer (depending on year) and then moved southward (decreasing values) and were at their most southern positions in October of each year (Fig. 5a). We observed significant differences in the location of sharks relative to their tagging site between months of the year (F_11, 550_ = 4.47, p<0.001), in which sharks were further north in March, April, and May than in September, October, and November (Fig. 5b; p<0.05 for all pairwise comparisons). For sharks tagged at Three Tree Point, directed movement from summer to fall occurred from the region of Alki to the regions of Three Tree Point and Robinson Point, respectively (Fig. 1), a difference of 10–25 km between the seasons. Sharks tagged in Elliott Bay did not move as far, but still moved from inside Elliott Bay in the spring and summer to areas in the main channel near Alki in the fall and winter, a difference of ∼7–10 km. Males were generally further south of their tagging site than females (F_1, 550_ = 7.50, p = 0.006; −5.5 km and −1.01 km, respectively) and larger sharks were generally further north of their tagging site than smaller sharks (F_1, 550_ = 12.54, p<0.001; distance from tagsite = 0.089 *TL – 19.022). All interactions were non-significant (p>0.25 for all interactions except gender*month (F_11, 550_ = 1.39, p = 0.176)) and shark was a non-significant random effect (z = 0.68, p = 0.247).

**Figure 6:**
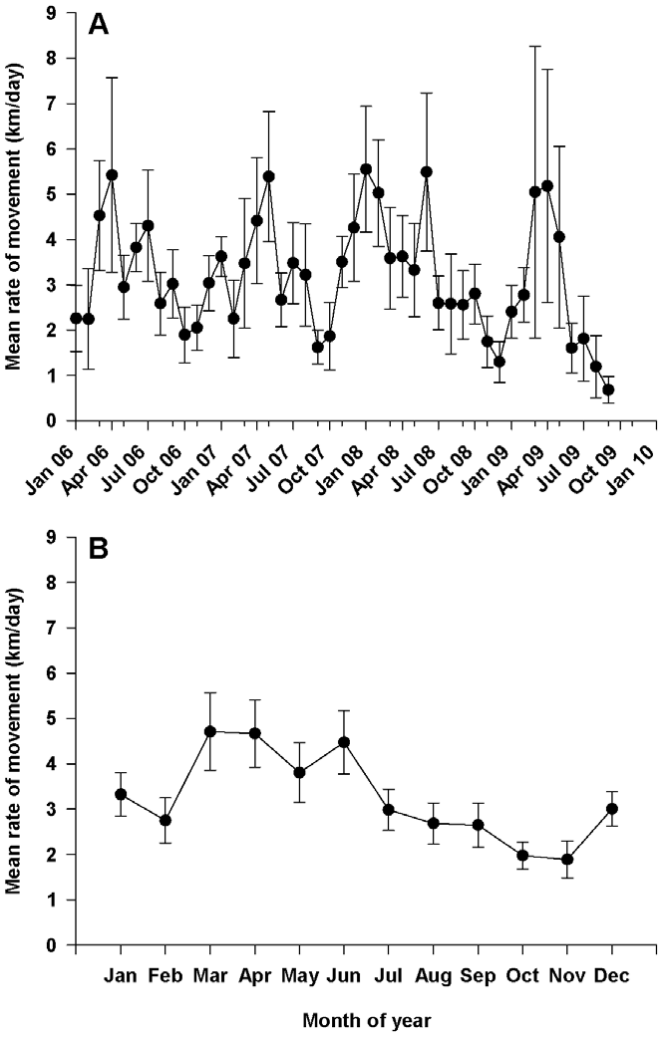
Mean rate of movement (km/day) of sixgill sharks in Puget Sound, WA, USA. Values were calculated across all sharks for A) each month from 2006 to 2009, and for B) each month across all years.

Mean rates of movement for sixgill sharks in Puget Sound were more variable over time than what we found for location of sharks (Fig. 6a). Our analysis found a significant TL*month interaction (F_11, 518_ = 2.56, p = 0.003) which was due to one month (June) in which there was a positive relationship between TL and mean rate of movement, whereas there was no relationship in all other months. Without this interaction term, there is a significant difference in the rate of movement between months (Fig. 6b; F_11, 541_ = 2.52, p = 0.004), while TL (F_1, 541_ = 2.97, p = 0.085) and gender (F_1, 541_ = 1.05, p = 0.306) were non-significant. However, multiple comparison tests between months did not reveal any significant differences at α level = 0.05 (March, April, and June were different from October at α level = 0.07). All other interactions were non-significant (p>0.25) and removed from the final model.

### Ontogenetic shift out of Puget Sound

Nineteen out of the 34 sixgill sharks left Puget Sound and were detected in the Strait of Juan de Fuca or Strait of Georgia. These large-scale movements occurred mostly during the spring (11 sharks) compared to winter (5 sharks), summer (2 sharks) or fall (1 shark). Sixteen of the sharks moved through the Strait of Juan de Fuca at some point, while only three migrated exclusively to and/or through the Strait of Georgia (Table 2). Most sharks were detected for short periods (1–2 hours) in the Straits of Juan de Fuca and Georgia, while three sharks (# 79, 83, & 203) moved in and out of detection range for 2–3 months. Four sharks (# 27, 89, 149, & 202) were detected at a second line on the outer coast as far south as Point Reyes, CA (>1500 km from its tagging location) and as far north as Queen Charlotte Strait, BC, Canada (>530 km from its tagging location).

**Table 2:** Detections of sixgill sharks outside Puget Sound by POST* acoustic receiver lines.

Shark ID	Destination*	Dates of detection at destination	Duration*	Distance*	RoM*
25	SOG	8 Jun 2008	1 hr	261	7
27	JDF	23 May 2008	0.5 hr	118	2
	Pt. Reyes, CA	20 Oct 2008	7 hrs	1267	8
28	JDF	8 May 2008	1 hr	109	2
32	JDF	14 May, 11 Jun, 16 Jun 2006	1–2 hrs each	144	3
	Howe Sound	30 Jun 2006–27 Aug 2006	Daily	72	5
	SSOG	13 Oct 2006	5 hrs	.	.
	Howe Sound	26 Mar 2007–23 July 2007	Daily	.	.
	Puget Sound	1 Nov 2007–27 Dec 2007	Daily	199	2
	JDF	30 Dec 2007	2 hrs	126	35
79	SOG	1 Apr 2008–22 May 2008	Sporadic	235	19
	Howe Sound	13 Jun 2008–14 Jun 2008	Daily	83	4
	Puget Sound	22 Jun 2008–27 Jun 2008	Daily	178	22
	JDF	30 Jun 2008	0.5 hr	105	33
81	JDF	20 Apr 2007	0.5 hr	147	18
83	JDF	8 Apr 2008–22 Jun 2008	Sporadic	118	10
86	JDF	22 Apr 2009	1 hr	126	26
89	JDF	14 Nov, 22 Nov, 3 Dec 2007	1 hr	118	2
	Lippy Point	15 Dec, 28 Dec 2007	1 hr	397	34
90	JDF	17 May 2008	1 hr	118	2
148	JDF	24 Feb 2009	0.5 hr	129	5
149	JDF	2 Jul, 15 July 2008	1 hr each	105	12
	Puget Sound	19 Jul 2008–4 Mar 2009	Daily	105	24
	JDF	9 Mar 2009	1 hr	126	26
	Willapa Bay	17 Mar 2009	0.5 hr	293	35
190	JDF	28 Mar 2007	3 hrs	120	2
195	SOG	16 May, 7 Jul 2008	1–2 hrs each	261	13
	JDF	25 Aug 2008	1 hr	156	3
202	SOG	8 Jul 2006	1 hr	285	5
	QCS	19 Jul 2006	0.5 hr	246	22
203	JDF	28 Feb 2007–3 May 2007	Sporadic	166	5
	SOG	13 Dec 2007	1 hr	156	1
1031	SOG	18 Mar 2008	1 hr	274	16
1035	JDF	21 Jun 2008	1 hr	105	2
4083	JDF	28 Mar 2008	1 hr	118	6

*POST: Pacific Ocean Shelf Tracking; Destination: POST line where sixgill was detected after leaving Puget Sound; Duration: amount of time spent at each destination; Distance: distance (km) moved from previous location; RoM: rate of movement from last Puget Sound detection or the previous POST location (km/day). SOG: Strait of Georgia; JDF: Strait of Juan de Fuca; QCS: Queen Charlotte Strait; SSOG: Southern Strait of Georgia. See Fig. 1 for location of POST lines.

**Figure 7:**
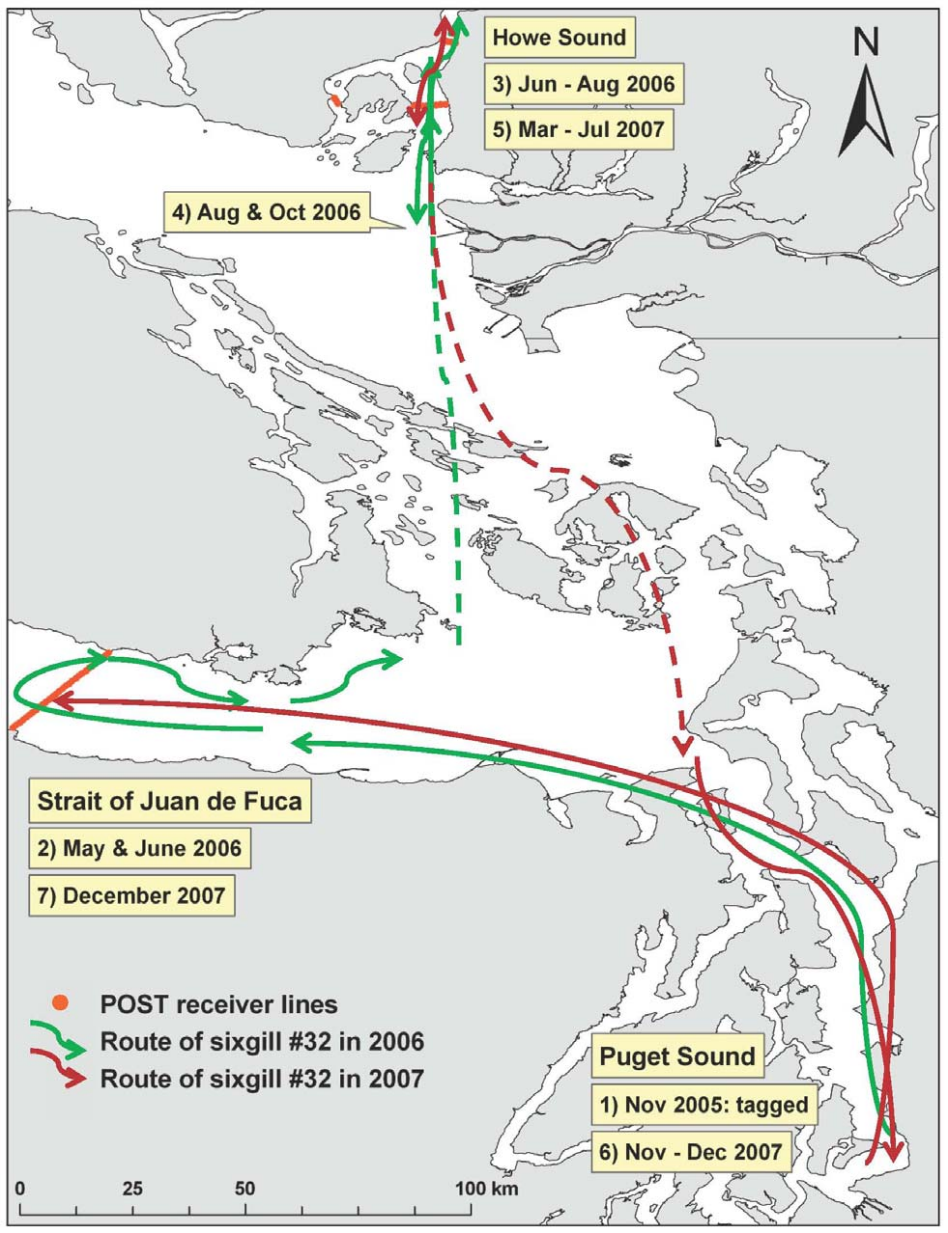
Movement of a female sixgill shark (#32) from 2005 to 2007.

In addition to sharks leaving Puget Sound and moving through the Straits of Juan de Fuca and Georgia, three sharks migrated back into Puget Sound at various time intervals (Table 2). Sharks # 32 & 79 came back to Puget Sound for short periods of time, while # 149 came back and stayed in Puget Sound for 8 months. We have the greatest details of behavior outside of Puget Sound on shark # 32 (Fig. 7), which was tagged in November 2005, left Puget Sound in April 2006, was detected 144 km away in the Strait of Juan de Fuca in May 2006 and then again in June 2006, moved to the Howe Sound, British Columbia region by June 2006 and remained in this general area until July 2007 before migrating back into Puget Sound in November 2007 for 2 months. This shark then left Puget Sound a second time and was detected in the Strait of Juan de Fuca for the second time at the end of December 2007.

**Figure 8:**
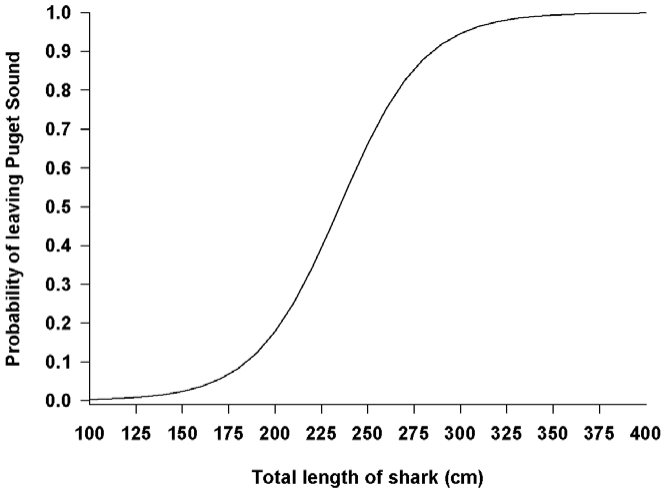
Probability of a female sixgill shark leaving Puget Sound, WA, USA as a function of the shark's total length.

Using the calculated final TL of a shark, we could predict whether females left Puget Sound and moved through the Strait of Juan de Fuca or Strait of Georgia better than by chance alone (χ^2^ = 6.95, df = 1, p = 0.008; rho-squared = 0.27), and calculated TL was a significant predictor of females leaving Puget Sound (Wald statistic = 2.04, p = 0.042). The odds ratio for female calculated TL was 1.045 (95% CI = 1.090 to 1.002) which suggested that for every 1-cm increase in TL of female sixgills, the probability of leaving Puget Sound increased by 4.5% (Fig. 8). Thus, we would be correct 50% of the time if we predicted that a 235-cm sixgill shark would leave Puget Sound this year. Although males also left Puget Sound and were detected in the Strait of Juan de Fuca and the Strait of Georgia, we could not predict whether males would leave Puget Sound based solely on calculated TL (χ^2^ = 1.12, df = 1, p = 0.289; Wald statistic = 1.01, p = 0.315).

We also detected a change in behavior of sharks prior to and during these large-scale moves out of Puget Sound. First, sharks moved more than twice as fast inside Puget Sound during the three-month period prior to each shark leaving Puget Sound (mean ± SD = 155±37 body lengths/hour) than during the same three-month period in the previous year when they did not leave (mean ± SD = 67±32 body lengths/hour) (paired t-test: t = −6.03, df = 6, p<0.001). Second, the rates of movement from Puget Sound to POST locations and the rates of movement between POST locations (Table 2) were generally higher than the rates of movement we observed within Puget Sound (Fig. 6a).

## Discussion

In this study, we were able to blend information from two large-scale passive acoustic arrays in order to monitor the movements of 34 sixgill sharks for nearly four years within Puget Sound and across more than 2000 km of the North American Pacific coast. Using these data, we found that individual sub-adult sixgill sharks displayed two distinct behavioral syndromes: 1) residency in Puget Sound, and 2) ontogenetic shifts to coastal waters.

### Residency in Puget Sound

Sixgill sharks were monitored for up to 1414 days inside Puget Sound waters after being tagged with acoustic transmitters. While resident in the Sound, sixgills were relocated at the same site as their previous detection more than 60% of the time and were relocated within the same region more than 70% of the time. Moreover, the parabolic relationship between sedentariness and the time between detections suggests an annual pattern in which sixgills are sedentary at specific locations for weeks at a time, move slowly away to a second core area after about six months, and then return to the original core area at the same time each year. This behavior supports a hypothesis that juvenile sixgills have a high degree of site fidelity, as seen with juveniles of other shark species [27], [66], [67]. This behavior also suggests that populations of sixgill sharks may be susceptible to localized human impacts such as fishing or pollution. Thus, restricted movement can exacerbate such life history traits as long generation times, long life spans, slow growth and low fecundity that are well known to make sharks vulnerable to anthropogenic threats [68]. The exploitation of shark species has been implicated in numerous studies explaining sharp declines in shark populations all over the world [e.g. 69], [70], [71]. We have little quantitative information on the size of sixgill populations in Puget Sound making the assessment of the risk from exploitation difficult; however, it is likely that sixgills are similar to other sharks with low productivity life-histories and could not withstand high levels of exploitation.

Seasonally, sixgills made consistent movements to the north from winter to spring and movements to the south from summer to fall. Sharks tagged at Three Tree Point spent most of their summers near Alki and then moved south to the Robinson Point region in the winter. Sharks tagged in Elliott Bay spent most of their summers inside Elliott Bay and then moved out into the main channel near Alki during the winter. In general, these similar north/south seasonal movements kept tagging groups separated during the year, although sixgills from different tagging groups have been tracked simultaneously at the same place (southern end of Bainbridge Island) during the summer [34]. The mechanisms responsible for the shift in habitat between spring and fall are unknown, but animals tend to move to forage, to escape predation, for thermoregulation advantages, or for reproductive or social interactions [e.g. 2], [15], [20], [23], [72]. Sixgills are the largest resident fish in Puget Sound and we have collected and actively tracked small (<200 cm) and large (>250 cm) sixgills in the same location at the same time, so it is unlikely that individuals are shifting habitats to reduce predation risk or that sixgills are segregated by size in any way to avoid predation from larger sixgills. Sixgills do move into shallower water in the spring [34], coinciding with seasonal movements, but they are still in waters deeper than 100 m and the temperature of Puget Sound is relatively constant at depths below 100 meters [34], [73]; thus, thermoregulation seems an unlikely mechanism as well. The relatively small magnitude of seasonal movement does not suggest they are related to reproductive or mating behavior, and we did not observe interactions between gender and month in any of our analyses that would suggest there were differences in behavior between males and females at different times of the year. Thus, it seems most plausible that sixgills are following some seasonal shift in prey resources.

In Puget Sound, much of the food web is driven by the seasonal production of phytoplankton and macroalgae which begin to bloom in the spring [50], [51] and the diets of benthic fishes vary seasonally [74]. In fact, one-third of the benthic fishes in Puget Sound switch prey guilds between summer, fall, and winter; converging on abundant prey populations in the summer and diverging to more niche-specific prey in the winter [74]. Diet of sixgills in Puget Sound is unknown, but it is reasonable to expect sixgills to move towards and forage on seasonally abundant prey resources similar to other benthic fishes in Puget Sound [74] or similar to other large predators in other systems [e.g. 75], [76], [77].

### Ontogenetic shift to coastal waters

After residing in Puget Sound for up to four years after tagging, sixgills left and generally migrated through the Strait of Juan de Fuca for the outer coast. Prior to leaving Puget Sound, we observed a marked change in the behavior of sixgills: rates of movement were twice as fast during the three months prior to leaving compared to the same three months in previous years. However, this change may not represent an actual increase in the speed sixgills are moving, but may instead be the result of making more directed moves between sites. During 24-hour active tracking sessions [Levin et al. unpublished data], sixgills occupied small areas (<3 km^2^) and had highly tortuous paths. Thus, as sixgills age, movement may become more directed. Regardless of the mechanism, this was a definitive change in behavior leading up to their exodus from Puget Sound.

Movement of sixgills out of Puget Sound was explained by the shark's total length for females but not males. As females reached sizes >235 cm they had a greater probability (50%) of leaving. Most sixgills left Puget Sound during the spring and were detected moving through the Strait of Juan de Fuca or Strait of Georgia. Some moved between the two straits and a few were detected on the outer coast as far north as the Queen Charlotte Islands, BC, Canada and as far south as Point Reyes, CA, USA. Interestingly, three sharks returned to Puget Sound after being away for days or years. These sixgills were all females. Indeed, one individual (# 32) returned to Puget Sound after being away for nearly two years. This movement may be related to reproductive cycles or mating behavior. It is thought that sixgills have two-year gestation periods, similar to another Puget Sound shark species, the spiny dogfish *Squalus acanthias* [78]. However, according to our calculations of growth, this female was ∼262 cm when it returned to Puget Sound in November 2007 (225 cm in November 2005). This is well below the size-at-maturity estimate for females at ∼420 cm [55]. Our growth calculations may be too coarse and this particular shark may have grown faster and been much closer to size-at-maturity; but, it seems likely these sharks returned to Puget Sound for reasons other than reproduction (e.g. following prey resources).

### Conclusions

We monitored the patterns of movement of sixgill sharks for nearly four years and found consistent patterns of movement while they were residents and a distinct change in behavior as they left Puget Sound. For most large marine predators, we have a limited understanding of how they move through their environment, and this clouds our ability to successfully manage their populations and their communities. With detailed movement information, we can begin to quantify the spatial and temporal impacts of and on large predators within the framework of their ecosystems.
